# Efficacy of the Osaka Medical College (OMC) brace in the treatment of adolescent idiopathic scoliosis following Scoliosis Research Society brace studies criteria

**DOI:** 10.1186/s13013-015-0036-9

**Published:** 2015-04-11

**Authors:** Hiroshi Kuroki, Naoki Inomata, Hideaki Hamanaka, Kiyoshi Higa, Etsuo Chosa, Naoya Tajima

**Affiliations:** Department of Orthopaedic Surgery, National Hospital Organization Miyazaki Higashi Hospital, 4374-1 Tayoshi Ooaza, Miyazaki, 880-0911 Japan; Department of Orthopaedic Surgery, University of Miyazaki Faculty of Medicine, Miyazaki, Japan; Department of Orthopaedic Surgery, Nozaki Higashi Hospital, Miyazaki, Japan

**Keywords:** Adolescent idiopathic scoliosis (AIS), Osaka Medical College (OMC) brace, Conservative treatment, Hanging total spine x-ray, Standardized inclusion and assessment criteria, Scoliosis Research Society (SRS)

## Abstract

**Background:**

The efficacy of brace treatment for patients with adolescent idiopathic scoliosis (AIS) remains controversial. To make comparisons among studies more valid and reliable, the Scoliosis Research Society (SRS) has standardized criteria for brace studies in patients with AIS. The purpose of this study was to evaluate the efficacy of the Osaka Medical College (OMC) brace for AIS in accordance with the modified standardized criteria proposed by the SRS committee on bracing and non-operative management.

**Methods:**

From 1999 through 2010, 31 consecutive patients with AIS who were newly prescribed the OMC brace and met the modified SRS criteria were studied. The study included 2 boys and 29 girls with a mean age of 12 years and 0 month. Patients were instructed to wear the brace for a minimum of 20 hours per day at the beginning of brace treatment. The mean duration of brace treatment was 4 years and 8 months. We examined the initial brace correction rate and the clinical outcomes of main curves evaluated by curve progression and surgical rate, and the compliance evaluated by the instruction adherence rate for all cases. The clinical course of the brace treatment was considered progression if ≥6° curvature increase occurred and improvement if ≥6° curvature decrease occurred according to SRS judgment criteria.

**Results:**

The average initial brace correction rate was 46.8%. In 10 cases the curve progressed, 6 cases the curve improved, and 15 cases the curve remained unchanged (success rate: 67.7%). The mean instruction adherence rate, that was defined the percentage of the visits that patients declared they mostly followed our instruction to total visits, was 53.7%. The success rate was statistically higher in the patient group whose instruction adherence rate was greater than 50% (88.2%) as compared with in those 50% or less (42.8%).

**Conclusions:**

OMC brace treatment for AIS patients could alter the natural history and significantly decreased the progression of curves to the threshold for surgical intervention. Better instruction adherence of brace wear associated with greater success.

## Introduction

Operative treatment for adolescent idiopathic scoliosis (AIS) has been rapidly progressed as a result of the development of the spinal instrumentation that makes possible three-dimensional correction of spinal deformity [1-3]. However, the role of non-operative treatment is still indispensable for patients with mild to moderate AIS. Although in the past, numerous non-operative methods, including physical therapy, exercise, massage, manipulation, and electrical stimulation, have been attempted, brace treatment is generally perceived as the only potentially effective in preventing curve progression and the subsequent need for surgery [4,5]. Various types of brace have been invented and practically used for more than 50 years [6].

**Figure 1:**
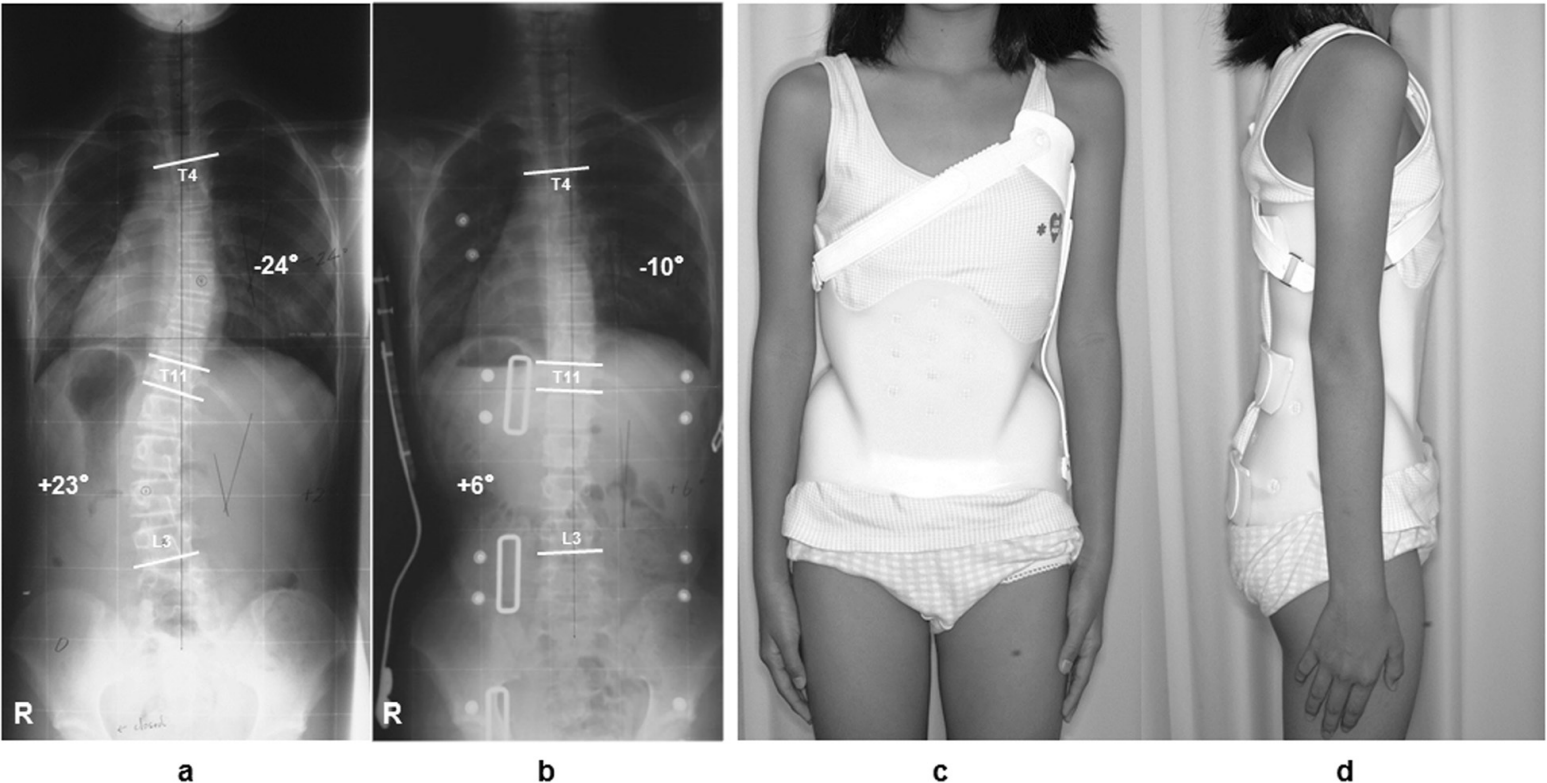
**Osaka Medical College (OMC) brace.** The OMC brace satisfactorily corrects double major curves **(a,** **b)** and a standing posture in brace is well balanced **(c,** **d)**.

We usually use the Osaka Medical College (OMC) brace for the treatment of AIS patients who meet some requirements as follows; still under growing, a Cobb angle of between 25 and 50°, and an apex of caudad to T7, with expectation to halt the progression of curve and to improve cosmetic appearance. The OMC brace is one of the popular custom-made thoracolumbosacral orthoses (TLSOs) in Japan developed by Onomura in 1970′s [7]. The characteristics of the OMC brace are represented by inconspicuous design, light weight, reduction of restriction on the chest wall movement, and ability to correct the high thoracic curve by righting reflex [7]. The concept of this brace is maintenance of whole body alignment and balance. For the achievement of these goals, step-by-step molding from pelvic girdle to high thoracic level with correcting lumbar and main thoracic curves is important to generate desirable corrective force based on the principle of three points lateral compression [8] (Figure 1).

Whereas, the efficacy of brace treatment for AIS continues to be controversial, with some authors reporting control of curve progression with bracing and others reporting that bracing fails to alter the natural history [9]. The lack of consistency of both the inclusion criteria and the definitions of brace effectiveness makes many clinicians skeptical about the efficacy of brace treatment [10].

To make comparisons among studies more valid and reliable, the Scoliosis Research Society (SRS) has standardized criteria for brace studies in patients with AIS [11].

The purpose of the present study was to evaluate the efficacy of the OMC brace for AIS patients in accordance with the new standardized criteria proposed by the SRS committee on bracing and non-operative management. The current report is the first detailed description focused on efficacy of OMC brace treatment for Japanese AIS patients based on SRS criteria.

## Materials and methods

### Study overview

Medical records and plain x-rays of patients with AIS who were treated with the OMC brace were retrospectively reviewed. To be included in this study, patients met the following criteria: diagnosis of AIS with radiological confirmation of absence of significant pathological malformation of the spine, age 10 years and older when their brace was prescribed, Risser 0–2, primary curve magnitude from 20 to 40°, no prior treatment, and if female, either premenarchal or <1 year postmenarchal. Although primary curve magnitude from 25 to 40° was expressed in SRS criteria, it was our treatment protocol to include immature patients who have progressive scoliosis from 20 to 40°, as a result we employed patients with Cobb angles from 20 to 24°, which is in accordance with a principle of Weinstein et al. [12]. Further, in SRS criteria, only patients who completed their brace treatment and were followed for more than 2 years after skeletal maturity were included. However, Negrini et al. [13] reported the fact that only 85% of patients reached the 2 years follow-up, but this subgroup was not different from the entire population for any basal characteristic nor any final results in their study. For this reason, we employed a follow-up of within 2 years beyond skeletal maturity to increase the number of patients available.

### Patient population

From 1999 through 2010, 31 consecutive patients with AIS who were newly prescribed the OMC brace were studied. The study included 2 boys and 29 girls ranging in age from 10 years and 10 months to 14 years and 8 months, with a mean age of 12 years and 0 month. The type of curves consisted of thoracic in 4 cases, thoracolumbar in 4 cases, lumbar in 12 cases, double major in 7 cases, double thoracic in 1 case, and triple major in 3 cases. Risser stage was grade 0 in 20 cases, grade 1 in 5 cases, and grade 2 in 6 cases. Apexes of main curves were all lower than T7 (T8 in 5 cases, T9 in 6 cases, T10 in 3 cases, T11 in 1 case, T12 in 3 cases, L1 in 1 case, L2 in 8 cases, and L3 in 4 cases). The mean pre-brace Cobb angle of main curves was 27.3°, with the range from 21° to 36°. The duration of brace treatment was from 3 years and 1 month to 6 years and 10 months, with a mean period of 4 years and 8 months. Of these, during brace wear follow-up times were from 1 year and 10 month to 6 years and 1 month, with a mean period of 3 years and 4 months. And post brace weaning follow-up times were from 0 month to 3 years and 3 months, with a mean period of 1 year and 4 months.

### Data collection and analysis

We examined the initial in brace correction rate at the brace prescription, the clinical outcomes of main curves evaluated by curve progression and surgical rate, and the compliance evaluated by the instruction adherence rate for all cases. The estimated number of hours of brace wear in each patient was monitored by self-statement. And the instruction adherence rate was calculated to express the compliance. Curve flexibility was recognized by the hanging correction rate utilized hanging total spine x-ray [8]. The brace correction rate, the hanging correction rate, and the instruction adherence rate were conducted by formulae as described below.

- Brace correction rate (%) = {(Cobb angle in upright position - Cobb angle on initial brace wear)/Cobb angle in upright position} × 100.
- Hanging correction rate (%) = {(Cobb angle in upright position - Cobb angle in hanging position)/Cobb angle in upright position} × 100.
- Instruction adherence rate (%) = (number of times to visit the outpatient clinic when the patient declared that he or she could wear the brace more than instructed hours minus 2 hours/all number of times to visit the outpatient clinic) × 100.

The clinical outcome was assessed based on the SRS criteria. According to the Cobb angle on standing anteroposterior spine x-rays that made with the patients out of the brace were classified as: (1) improved: decrease of the Cobb angle by 6° or more, (2) stable: no more than 5° of progression or improvement, (3) progressed: increase of the Cobb angle by 6° or more, and (4) progression beyond the Cobb angle of 45° who were considered candidates for surgery. All radiographic measurements were made by 1 author using the same protractor to minimize inter-observer variability in accordance with a concept of Lee et al. [6].

Statistical analyses were defined using a two-tailed paired *t*-test and chi square test. A value of P < 0.05 was considered statistically significant.

### Brace management protocol

All OMC braces were fabricated by the same certified orthotist. A plaster cast was taken to capture the body shape of each patient, which was used by the certified orthotist to custom make each OMC brace. Then, not only the optimal correction but also comfortable fit and function consisted of pressure pad placements were ensured. Also, standing anteroposterior and lateral spine x-rays were used to check the in brace correction and whole spinal alignment including pelvis while the brace was being worn.

Patients were instructed to wear the brace for a minimum of 20 hours per day at the beginning of brace treatment. When skeletal maturity was noted, that is, all of the following three criteria were fulfilled; a Risser stage of 4, at least 2 years passed since the onset of menstruation (for girls), two consecutive visits over a time period of at least 1 year with no more than a 1-cm increase in height, the brace weaning started and the time in brace was slowly reduced during 1 year. Finally the brace wearing was stopped at 1 year post skeletal maturity.

### Consent

The study was approved by the University’s Institutional Review Board and written informed consent was obtained from the patients and their parents prior to participating. And all procedures were in accordance with the Helsinki declaration.

## Results

**Figure 2:**
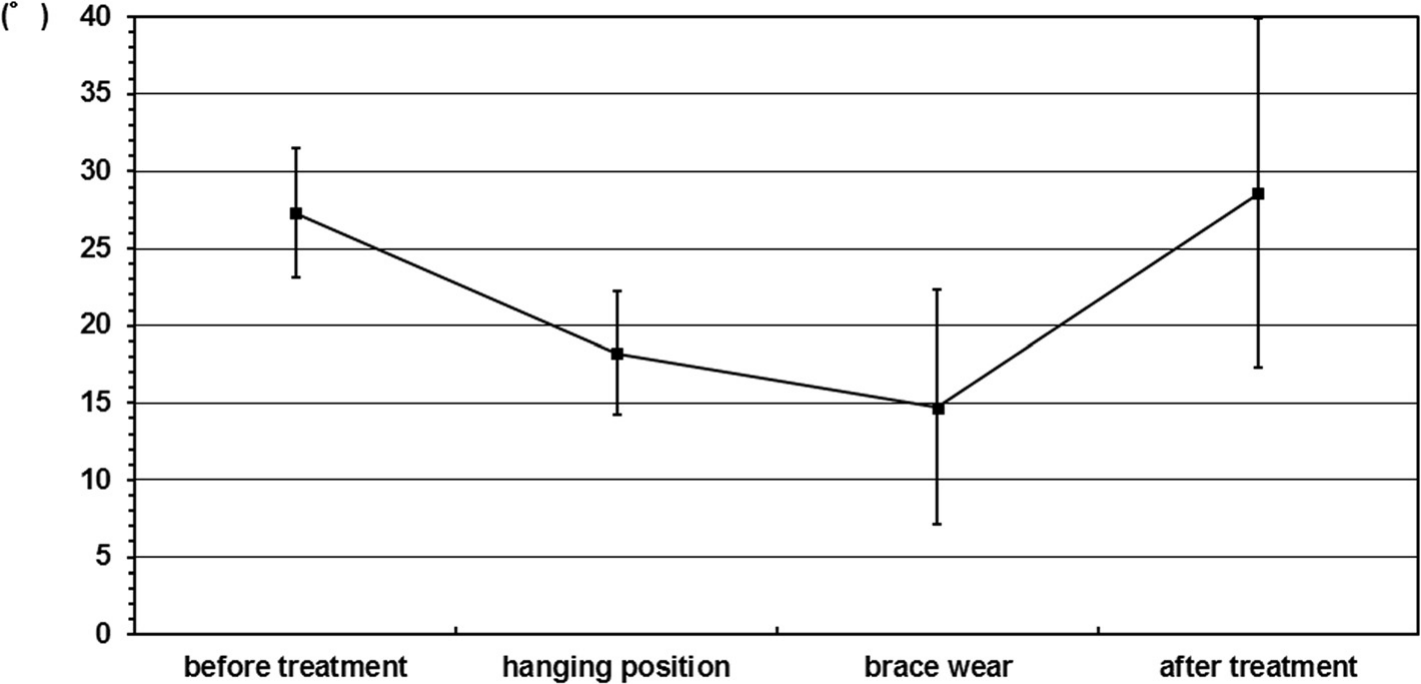
**Sequential changes of the Cobb angle.** The average Cobb angles in upright position before treatment, in hanging position, on initial brace wear, and at final follow-up were 27.3 ± 4.2°, 18.2 ± 4.0°, 14.7 ± 6.7°, and 28.6 ± 11.3°, respectively. The main curves were corrected by wear of the OMC brace better than hanging position. And OMC brace treatment could prevent the progression of curves during periods of growth.

The average Cobb angles in upright position before treatment and at final follow-up were 27.3 ± 4.2° and 28.6 ± 11.3°, respectively. As a result, the OMC brace could prevent curve progression during periods of growth (Figure 2). The average in brace Cobb angle on initial brace wear was 14.7 ± 6.7° and the average brace correction rate was 46.8 ± 25.1%. Meanwhile, the average Cobb angle in hanging position was 18.2 ± 4.0° and the average hanging correction rate was 33.0 ± 14.1%. The average brace correction rate was statistically better than the average hanging correction rate.

**Figure 3:**
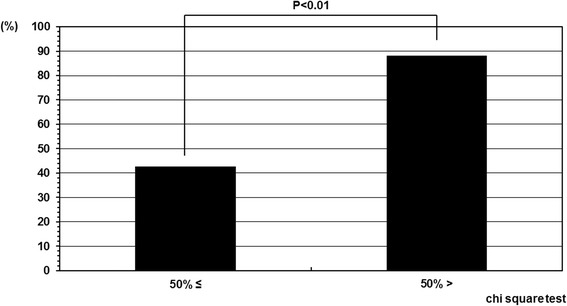
**Success rate depend on compliance.** The success rate in the patient group whose instruction adherence rate was greater than 50% and 50% or less were 88.2% and 42.8%, respectively. The success rate was statistically higher in the patient group whose instruction adherence rate was greater than 50% as compared with in those 50% or less.

At final follow-up, the curve progressed in 10 cases, the curve improved in 6 cases, and the curve remained unchanged in 15 cases. The success rate of 67.7% (21/31) was achieved although the average instruction adherence rate was 53.7%. Further, only 3 cases out of 31 progressed beyond the Cobb angle of 45° were considered candidates for surgery. Therefore, the rate for progression to surgical indication was 9.7% (3/31). However, the cases were divided in two groups according to the instruction adherence rate, and as a result, the success rate was statistically higher in the patient group whose instruction adherence rate was greater than 50% compared with in those 50% or less. The success rate in the patient group whose instruction adherence rate was 50% or less was 42.8%. On the other hand, the success rate in the patient group whose instruction adherence rate was greater than 50% was 88.2% (Figure 3). And the instruction adherence rates of 3 cases whose Cobb angles progressed beyond 45° were all 50% or less (0%, 0%, and 42.9%).

### Illustrative cases presentation

#### Case 1: 12 years 10 months girl

**Figure 4:**
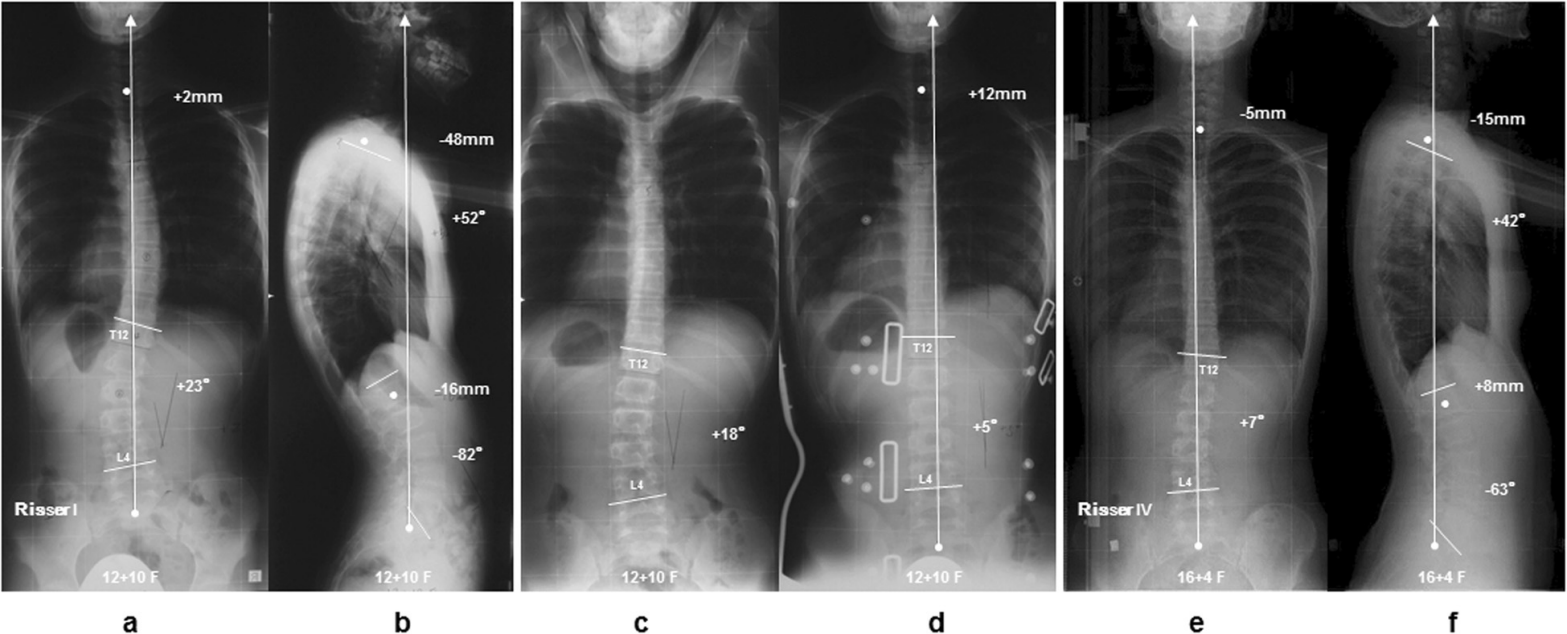
**Case 1: 12 years 10 months girl.** The patient had a left convex 23° lumbar scoliosis before treatment **(a,** **b)**. The angles of lumbar curves were corrected to 18° in hanging position and 5° in brace wear, respectively **(c,** **d)**. Throughout brace treatment for 3 years and 5 months, her instruction adherence rate was maintained at 100%, and the Cobb angle improved to 7° at final follow-up **(e,** **f)**.

The patient presented a left convex 23° lumbar scoliosis before treatment. The Cobb angle of the lumbar curve in hanging position was 18°. An in-OMC brace x-ray of the patient was demonstrated 5° due to correction created by the pressure pad pushing the apex of L2 between T12 and pelvis. Her instruction adherence rate was maintained at the 100% throughout brace treatment for 3 years 5 months. At final follow-up, the Cobb angle improved to 7° (Figure 4).

#### Case 2: 11 years 3 months girl

**Figure 5:**
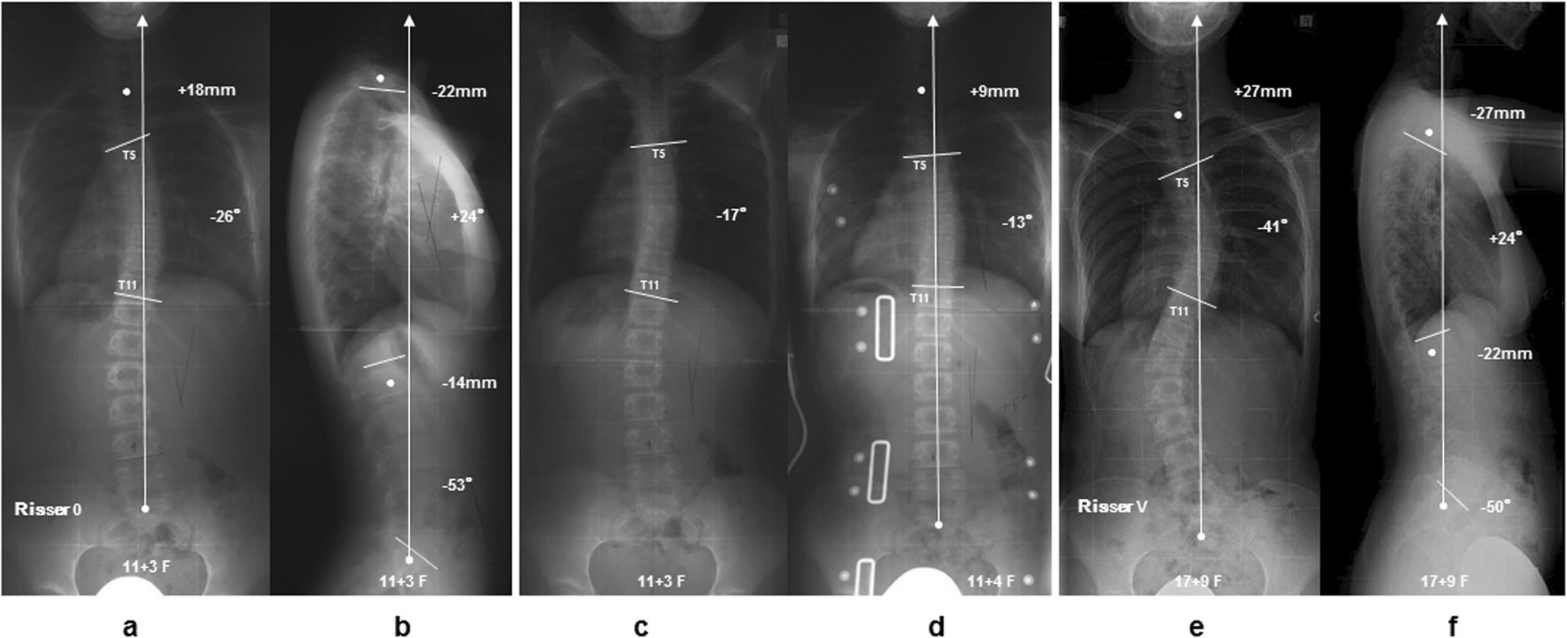
**Case 2: 11 years 3 months girl.** The patient had a right convex 26° thoracic scoliosis before treatment **(a,** **b)**. The angles of thoracic curves were corrected to 17° in hanging position and 13° in brace wear, respectively **(c,** **d)**. Throughout brace treatment for 6 years and 5 months, her instruction adherence rate remained at only 7.7%, and the Cobb angle progressed to 41° at final follow-up **(e,** **f)**.

The patient presented a left convex 26° thoracic scoliosis before treatment. The Cobb angle of the thoracic curve in hanging position was 17°. An in-OMC brace x-ray of the patient was demonstrated 13° due to correction created by the pressure pad pushing the apex of T8 between T5 and T11. Her instruction adherence rate was remained at the only 7.7% throughout brace treatment for 6 years 5 months. At final follow-up, the Cobb angle progressed to 41° (Figure 5).

## Discussion

Skeletally immature patients with AIS are at risk for curve progression together with growing. Thus, the main goal of brace treatment in AIS is to change the natural history by stopping curve progression. Brace treatment has been growing more and more important because the discovery rate of AIS in an early stage has been presently increased owning to the well-established periodic school screening [14]. Current recommendations from the SRS include the initiation of brace treatment in skeletally immature patients who present with curves greater than 30° on initial presentation or in patients who progress greater than 10° to a magnitude greater than 25° [15].

According to Lonstein and Carlson [16], 68% of patients with a Risser stage of 0 or 1 progressed greater than 5° for curve between 20° and 29°. Bunnell [17] also reported progression of at least 5° in 68%, 10° in 34%, and 20° in 18% patients in his series. Further, patients diagnosed before 10 years had an 88% risk of progression of 5° or more and this risk was the same for those diagnosed between the ages of 10 and 12. Weinstein et al. [18] followed curves for an average of 40 years and nearly 70% of curves measuring a minimum of 30° progressed after skeletal maturity. Similarly, Nachemson et al. [4] demonstrated 66% of observed patients with idiopathic scoliosis curves measuring 20° to 35° progressed 6°. Karol et al. [19] found 32% of boys presenting with a curve of at least 25° and all Risser stages progressed 10° or more. To be considered an effective management method, Janicki et al. [20] stated that a brace must prevent progression in at least 70% of patients with AIS. In this study, 67.7% patients achieved curve progression of less than 6° at the skeletal maturity. Further, only 9.7% patients reached Cobb angle of more than 45° which meant surgical indication. These results verified that OMC brace treatment could change the natural history of AIS just like as other TLSOs previously reported [6,10,13,20-25] (Table 1).

**Table 1:** **Literature review of the clinical results under SRS criteria**

**Author (Year)**	**Treatment period**	**Type of brace**	**Success rate**	**Progression rate for surgical indication**
Coillard C et al. (2007) [10]	?	SpiceCorBrace	59.4%^**^	24.1%^+^
Janicki JA et al. (2007) [20]	1y5m	TLSO	85.4%^*^	62.4%^+^
	1y4m	Providence	68.6%^*^	42.9%^+^
Negrini S et al. (2009) [13]	4y2m	Lyon, SPoRT	95.8%^**^	0.0%^+^
Aulisa AG et al. (2009) [21]	4y11m	Progressive Action Short Brace	100.0%^*^	0.0%^+^
Zaborowska-Sapeta K et al. (2011) [22]	2y8m	Chêneau brace	48.1%^*^	12.7%^++^
Lee CS et al. (2012) [6]	2y9m	Chaleston Bending Brace	84.2%^**^	12.6%^+^
Aulisa AG et al. (2012) [23]	3y6m	Progressive Action Short Brace	100.0%^*^	0.0%^+^
Weinstein SL et al. (2013) [12,24]	?	TLSO	_	28.1%^++^
Maruyama T et al. (2013) [25]	1y9m	Rigo-Chêneau brace	70.8%^**^	18.2%^+^
Kuroki H et al. (2015) [current report]	3y4m	Osaka Medical College Brace	67.7%^**^	9.7%^+^

^*^progression < 5°, ^**^progression < 6°, ^+^progression ≥ 45°, ++progression ≥ 50°.

With regard to ability of curve correction, the average initial in brace correction of the OMC brace was 46.8%. This was inferior to the Charleston bending brace but almost same as other TLSOs [26-36] (Table 2). Although the OMC brace is a TLSO, correction and controlling of the upper curve in double thoracic scoliosis could be achieved to utilize the righting reflex that was generated by the active bending for the high thoracic curve under bracing [7].

**Table 2:** **Literature review of the initial correction rate**

**Author (Year)**	**Apex**	**Type of brace**	**Correction rate**
Watts HG et al. (1977) [26]	below T10	Boston Brace	54.7%
Uden A et al. (1982) [27]	below T7	Boston Brace	41.0%
		Milwaukee Brace	10.0%
Jonasson-Rajala E et al. (1984) [28]	below T8	Boston Thoracic Brace	46.2%
		Boston Milwaukee Brace	29.3%
		Boston Brace	36.9%
Ohta K et al. (1988) [29]	-	Active Correction Brace	53.8%
Kawakami N et al. (1991) [30]	-	Active Correction Brace	17.6%
Asazuma T et al. (1991) [31]	below T7	Under Arm Brace	23.0%^*^
Arai S et al. (1992) [32]	-	Milwaukee Brace	44.2%^*^
Iwaya D et al. (1997) [33]	below T7	Charleston Bending Brace	75.0%
Semoto Y et al. (1999) [34]	below T7	OMC Brace	35.5%
Spoonamore MJ et al. (2001) [35]	-	Rosenberger Brace	30.0%
D’Amato CR et al. (2004) [36]	-	Providence Brace	96.0%
Kuroki H et al. (2015) [current report]	below T8	OMC Brace	46.8%

^*^maximum correction rate.

The amount of time spent in the brace during the active phase has been still under debate. Blount et al. [37] originally described full-time brace wear as 23 hours a day and Rowe et al. [5] found that bracing for 23 hours a day was the most successful means in their meta-analysis. Aulisa et al. [38] also stated that wearing the brace only overnight was associated with a high rate of curve progression. Indeed, the guidelines released by the Society on Scoliosis Orthopaedic and Rehabilitation Treatment (SOSORT) indicate adherence as a key element in determining the efficacy of bracing [15]. However, some other studies reported successful treatment with part-time brace wear as 16 hours a day [39,40]. Schiller et al. [41] stated part-time or nighttime bracing (Charleston, Providence) may be effective for curves less than 35°; however, curves greater than 35° often require full-time bracing to reliably limit curve progression. Weinstein et at [24] indicated brace wear for an average of at least 12.9 hours a day was associated with success rate of 90 to 93%. In our previous study, there was statistical difference between 7–12 hours and 13–18 hours a day but no statistical difference between 13–18 hours and 19–24 hours a day [42]. So, at least 13 hours in brace a day is basically supposed to be mandatory to halt progression of curves.

As the documented problems of brace treatment, compliance, drop out, and psychological stress have been previously emphasized and investigated by many authors. Especially, compliance appears to be the greatest concern for any treating physician and the primary cause for poor results from brace treatment. However, it is not so easy to keep brace wear as scheduled in most adolescent patients. Gepstein et al. [43] showed that in all cases, patients or their parents reported the Charleston bending brace was worn for at least 80% of the prescribe time, yet DiRaimondo et al. [44] stated that only 15% of patients were highly compliant. Nicolson et al. [45] also used temperature data loggers at the brace-skin interface to measure time in the brace and found patients overestimated their time in brace nearly 150%. In current study, the instruction adherence rate was only 53.7% even under self-statement. The drop out rates in the literatures were from 20 to 40% [46-52]. In our past study, the drop out rate was held about 18.5% [42]. With respect to the psychological stress, Climent et al. [53] told that the brace treatment showed an impact on the overall quality of life. Matsunaga et al. [54] showed the rate of patients with psychological problems increased from 7.6% to 82.1% at 1 month after the start of brace treatment. Further, MacLean et al. [55] mentioned about the psychological effects of brace wear treatment for not only the patients themselves but also their parents. These are truly key points for success of brace treatment. Therefore, it is important to relieve the emotional stress during brace treatment as much as possible by mental support for AIS patients on frequent and periodic consultation (once every 3 to 6 months depending on the condition of each case). Thereby, brace wear can be facilitated and the brace treatment will be maintained as scheduled.

There are several limitations in this study. First, this project was small sample size. Second long-term follow-up was missing. Third, compliance of the patients was low, that is, the average instruction adherence rate of our subject was only 53.7% although it was subjectively obtained due to the lack of an objective compliance monitor in the braces. A prospective long-term study with larger sample size consisted of sufficient compliant patients is suggested in order to draw a solid conclusion. However, we believe that our study will contribute some improvement for the future management of AIS because accumulation of these minor data based on clinical experiences from a great number of institutions must be essential to the future solution of issues regarding efficacy of brace treatment for AIS.

In conclusion, OMC brace treatment for AIS in the skeletally immature patients could alter the natural history and significantly decreased the progression of curves to the threshold for surgical intervention. Brace compliance was associated with greater success in this study. By a great deal of care for AIS patients at the periodic consultations, motivation for brace wear must be inspired and it will consequently lead to an acceptable clinical outcome, that is, not only physically but also emotionally balanced body with Cobb angle under 45°.
